# Metal-Free Heptazine-Based Porous Polymeric Network as Highly Efficient Catalyst for CO_2_ Capture and Conversion

**DOI:** 10.3389/fchem.2021.737511

**Published:** 2021-10-15

**Authors:** Neha Sharma, Bharat Ugale, Sunil Kumar, Kamalakannan Kailasam

**Affiliations:** Advanced Functional Nanomaterials, Energy and Environment Unit, Institute of Nano Science and Technology (INST), Mohali, India

**Keywords:** heptazines, porous organic polymers, heterogeneous catalyst, carbon dioxide sorption, cycloaddition of carbon dioxide

## Abstract

The capture and catalytic conversion of CO_2_ into value-added chemicals is a promising and sustainable approach to tackle the global warming and energy crisis. The nitrogen-rich porous organic polymers are excellent materials for CO_2_ capture and separation. Herein, we present a nitrogen-rich heptazine-based microporous polymer for the cycloaddition reaction of CO_2_ with epoxides in the absence of metals and solvents. HMP-TAPA, being rich in the nitrogen site, showed a high CO_2_ uptake of 106.7 mg/g with an IAST selectivity of 30.79 toward CO_2_ over N_2_. Furthermore, HMP-TAPA showed high chemical and water stability without loss of any structural integrity. Besides CO_2_ sorption, the catalytic activity of HMP-TAPA was checked for the cycloaddition of CO_2_ and terminal epoxides, resulting in cyclic carbonate with high conversion (98%). They showed remarkable recyclability up to 5 cycles without loss of activity. Overall, this study represents a rare demonstration of the rational design of POPs (HMP-TAPA) for multiple applications.

## Introduction

Carbon dioxide (CO_2_) has attracted significant attention as a greenhouse gas which is considered to be a major contributor for global warming and subsequent climate changes. (Aresta et al., 2014; Pera-Titus, 2014; Mukherjee et al., 2019). During the last 10 years, the mean annual absolute CO_2_ concentration has increased by 2.21 ppm per year (Van Humbeck et al., 2014). Furthermore, various human activities including burning of fossil fuels have been contributing for the increase in the concentration of CO_2_ in the atmosphere and have resulted in serious environmental issues, like extreme weather and acidification of oceans (Tiba and Omri, 2017; Ding et al., 2019). Furthermore, coal-based power plants are known to contribute about 45% of the overall CO_2_ emission, and it is important to capture carbon dioxide from flue gas before releasing it to the atmosphere. Recently, a global initiative has been taken on the urgent need to develop technologies and processes for the effective capture and sequestration/separation of CO_2_ from post-combustion effluents such as flue gas wastes. Hence, extensive research efforts are being carried out by researchers worldwide on the development of systems for efficient carbon dioxide capture and storage (CCS) (Kitagawa et al., 2004; Yaghi et al., 2003; D’Alessandro et al., 2010; Bui et al., 2018). In this regard, the currently employed amine-based liquid-phase absorption technique is considered to be costly and energy-intensive. On the other hand, the adsorption of carbon dioxide on porous solid supports has been considered as a promising viable technique for CCS application because of its low energy requirement. In this context, solid porous adsorbents such as zeolites and activated carbons have been investigated for their potential applications for CCS (Rubin et al., 2007; House et al., 2009). Recently, POPs have emerged as promising candidate materials for selective CO_2_ storage and separation applications (Makal et al., 2012; Roeser et al., 2012; Su et al., 2017). In this regard, several strategies have been employed for modulation of the pore surfaces to induce selective gas adsorption property in the framework by the incorporation of Lewis acidic/basic sites and immobilization of polar functional groups (–NO_2_, –NH_2_, –CONH_2_, –CF_3_, etc.) (Yue et al., 2020). On the other hand, CO_2_ has gained considerable interest as a renewable, non-toxic, inexpensive, and an abundant C1 building block for high-value chemicals and fuels (Sakakura et al., 2007; Chu, 2009; Jacobson, 2009). However, a great challenge for CO_2_ conversion under mild conditions is its thermodynamic stability and kinetic inertness (Lanzafame et al., 2014). In this direction, several strategies have been developed to convert CO_2_ into value-added chemicals by forming C-N, C-O, C-C, and C-H bonds (Lanzafame et al., 2014; Yu and He, 2015). Especially, the cycloaddition of CO_2_ with epoxides to generate cyclic carbonates has gained a special interest because of its 100% atom economy along with high yield and selectivity. Furthermore, cyclic carbonates find widespread industrial applications as precursors for polymeric materials and intermediates in the synthesis of fine chemicals (Sakakura and Kohno, 2009). A variety of homogeneous catalysts such as alkali–metal salts (Qu et al., 2012; Ma et al., 2016), transition metal complexes (Ema et al., 2014; Ma et al., 2016), and ionic liquids (Wang et al., 2015) have been employed for the catalytic cycloaddition of CO_2_ with epoxides. However, these systems suffer from the inherent limitations which prevent their widespread application. To overcome the limitations of the homogeneous catalysts, several heterogeneous catalysts such as metal oxides (Yasuda et al., 2005), zeolites (Kuruppathparambil et al., 2016), and functional polymers (Meng et al., 2014) have been utilized. However, most of these catalysts require either high temperature (>100°C) or high pressure of CO_2_, thereby increasing the cost.

Among other potential materials, heptazine-based graphitic carbon nitride has gained significant interest in recent years due to its structure stability and possession of rich diverse properties (Wang et al., 2009; Cao et al., 2015). The heptazine nucleus C_6_N_7_ consists of three fused *s*-triazine rings, as a captivating kind of building block with a π-conjugated system (Battula et al., 2018). Nevertheless, with their excellent structural property, heptazine-based compounds/frameworks are still in their infancy to be explored due to unavailability of reactive and soluble heptazine precursors (Van Humbeck et al., 2014; Das et al., 2017). In 2002, Kroke et al. summarized the structural property of the soluble and reactive heptazine precursor, that is, 2, 5, 8-trichloro-*s*-heptazine or cyameluric chloride, which embarked the development of heptazine-based precursors and polymeric frameworks for different applications (Kroke et al., 2002; Kailasam et al., 2013; Li et al., 2013; Kailasam et al., 2016; Bala et al., 2017; Bala et al., 2018; Sharma et al., 2018; Sharma et al., 2021). In recent years metal-free POPs and covalent organic frameworks have been considered as a potential candidate for CO_2_ sorption and conversion (Zeng et al., 2016; Oschatz and Antonietti, 2018). Therefore, designing a metal-free polymeric network shows robustness and excellent CO_2_ capture, and conversion efficiency is high in demand. CO_2_ capture is highly dependent on the presence of surface polar groups on POPs which influence CO_2_ uptake capacity through Lewis acid–base interactions (Li and Zhao, 2013; Xu et al., 2017). The heptazine framework with a high nitrogen content and quite unique structural property offers a suitable choice for CO_2_ gas capture and conversion. Therefore, it is very prompting to engineer heptazine-based microporous polymeric materials with a wide range of N-rich organic linkers and their affinities for further exploration toward CO_2_ capture and conversion (Wang et al., 2016; Buyukcakir et al., 2017). Although, heptazine-based microporous polymers manifest their application toward CO_2_ sorption, but still there is no report of heptazine-based microporous polymers used for the conversion of CO_2_ into cyclic carbonate. Herein, we report a heptazine-based microporous polymeric network HMP-TAPA showing a high surface area and CO_2_ sorption in this detailed study.

## Experimental Section

### Materials

Commercially available reagents were used in all reactions without further purification. Tris (4-amino phenyl) amine (TAPA) was purchased from Sigma-Aldrich Chemical Co. and used without further purification. All epoxides and the internal standard used for catalytic reactions were purchased from TCI chemicals and used without further purification. Trichloro heptazine (TCH) was synthesized using a previously reported procedure (Kailasam et al., 2013; Kailasam et al., 2016).

### Material Characterization

FT-IR (Fourier transform infrared) spectra of the samples were measured by using a Bruker Vertex FT-IR 70/80 spectrometer in the spectral range of 4,000–650 cm^−1^. An Elementar Vario MACRO cube elemental analyzer was used to carry out elemental analysis (i.e., C, H, and N). Thermogravimetric analysis (TGA) was carried out by a SHIMADZU DTG-60H analyzer under the N_2_ environment (flow rate of 30 ml/min) with the temperature ranging from 50 to 500°C (ramping rate of 10°C/min). A solid-state NMR experiment was performed on a JNM-ECA/ECX series using a 5-mm FG NMR tunable probe. The products of the catalytic reactions were identified, and the catalytic conversions were determined by ^1^H-NMR spectra using 1, 1′, 2, 2′-tetrachloroethane as an internal standard and recorded in CDCl_3_ on the JEOL JNM-ECS-400 spectrometer operating at a frequency of 400 MHz. X-ray diffraction (XRD) patterns were examined using a Bruker D8 Advance diffractometer equipped with a scintillation counter detector, with a Cu-K_α_ radiation (λ = 0.15418 nm, 2θ = 5─80^o^) source operating at 40 kV and 40 mA.

The N_2_ physisorption measurements were measured at 77 K on an Autosorb iQ3 instrument (Quantachrome). The CO_2_ physisorption measurements were carried out at 273, 281, and 298 K. Ultrapure (99.995%) N_2_, He, and CO_2_ gases were used for the adsorption–desorption measurements. Prior to adsorption measurements, the sample (∼100 mg) was evacuated at 393 K under vacuum (20 mTorr) for 10 h. The specific surface area of the sample was recorded from the N_2_ adsorption isotherm via the Brunauer–Emmett–Teller (BET) micropore assistant method (*p*/p_0_ < 0.1). Pore size distributions were obtained from N_2_ desorption isotherms, using the NLDFT method. However, the total pore volume was calculated by the amount of N_2_ adsorbed at p/p_0_ ∼ 0.9.

A temperature-programmed desorption (TPD) study was carried out by BELCAT II (BELSORB) equipped with a thermal conductivity detector to estimate the volume of absorbed CO_2_. Initially, the sample was pretreated at 250°C to remove any trapped solvents and moisture. The TPD profile of CO_2_ was recorded at the rate of 10 C min^−1^ ranging from 50 to 350°C under He flow.

### Synthesis of a Heptazine-Based Porous Polymer

Tris-(4-aminophenyl)amine (0.344 mmol) in 1,4-dioaxne (40 ml) solution was added to a dry round-bottom (RB) flask accompanied by the dropwise addition of diisopropylamine (DIPEA; 2.064 mmol). Furthermore, this homogeneous solution mixture was allowed to cool at 0°C. In another RB flask, a solution of trichloroheptazine (Supplementary Figure S1; 0.344 mmol) in 1,4-dioaxne (10 ml) was prepared and added to the aforementioned homogeneous solution mixture with constant stirring. The temperature of the reaction mixture was maintained at 0°C during dropwise addition and then for another 30 min. After that, it was allowed to stir at room temperature, followed by refluxing at 100°C under nitrogen for 72 h with constant stirring.

Thereafter, the solid residue was collected by filtration and washed with THF, methanol, and acetone, followed by 48-h Soxhlet purification with THF:methanol mixture. After 48 h, the vacuum-dried product was obtained with 73% yield. The elemental analysis of solvent-free samples, calculated%, C:H:N, is found to be C: 55.84, H: 5.066, and N: 27.81 wt%. FTIR (cm^−1^): 3350, 1641, 1510, 1480, 1360, and 800.

### Catalytic Cycloaddition Reactions

A 25 ml high-pressure glass reactor was used for the cycloaddition reactions of CO_2_ and various epoxides. The purified and activated HMP-TAPA catalyst was measured (10 mg) and transferred to the reactor. Then the epoxides were added at room temperature, and the reactor was slowly pressurized with CO_2_, flushed twice. Then the required pressure was obtained (0.1–0.6 MPa) for 12 h. After that, the mixture was stirred at a temperature range of 60–80°C for the required time. After catalytic reactions, the reactor was allowed to cool down to RT, the leftover CO_2_ gas was released slowly, and the catalyst was separated by centrifugation. The recovered catalyst after each catalytic reaction was collected and washed with DCM–methanol mixture three times and then finally with pure MeOH. This is followed by drying at RT, and the catalyst is reused for successive catalytic cycles. The catalytic conversions were determined using 1,1′,2,2′-tetra-chloroethane as an internal standard. Percentage conversion can be calculated by using the formula.

## Results and Discussions

### Synthesis Scheme of HMP-TAPA

**SCHEME 1:**
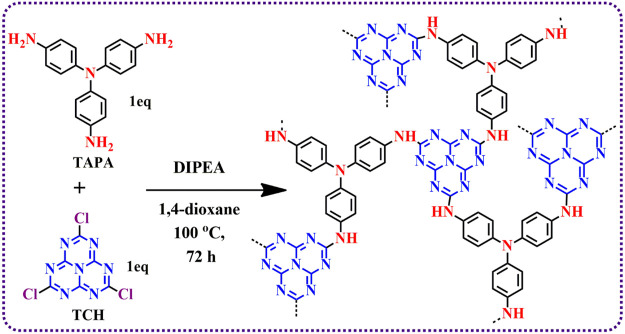
Synthesis of heptazine-based polymeric network HMP-TAPA.

To construct a heptazine-based polymeric network, TCH (a heptazine precursor) and Tris-(4-aminopenyl) amine were taken into an RB flask, which further underwent a substitution nucleophilic reaction in the presence of DIPEA (as the base) for 72 h. Scheme 1 shows the synthesis procedure of HMP-TAPA. Furthermore, purification was done by Soxhlet extraction using the THF:MeOH (1:1 ratio) mixture for 72 h, and after Soxhlet extraction, HMP-TAPA was vacuum-dried at 120°C before being used for further studies.

**GRAPHICAL ABSTARCT:**
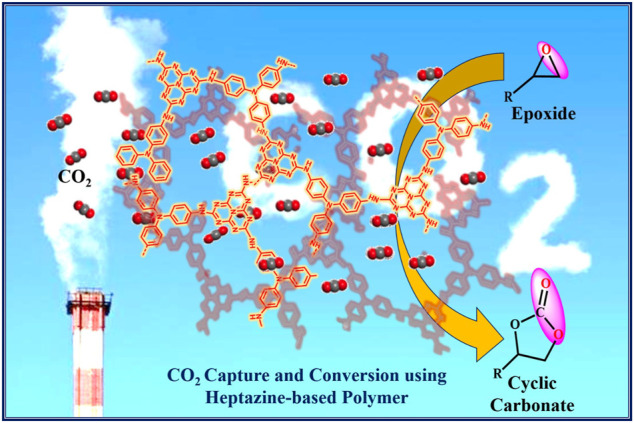


### Characterization of Catalysts

**FIGURE 1:**
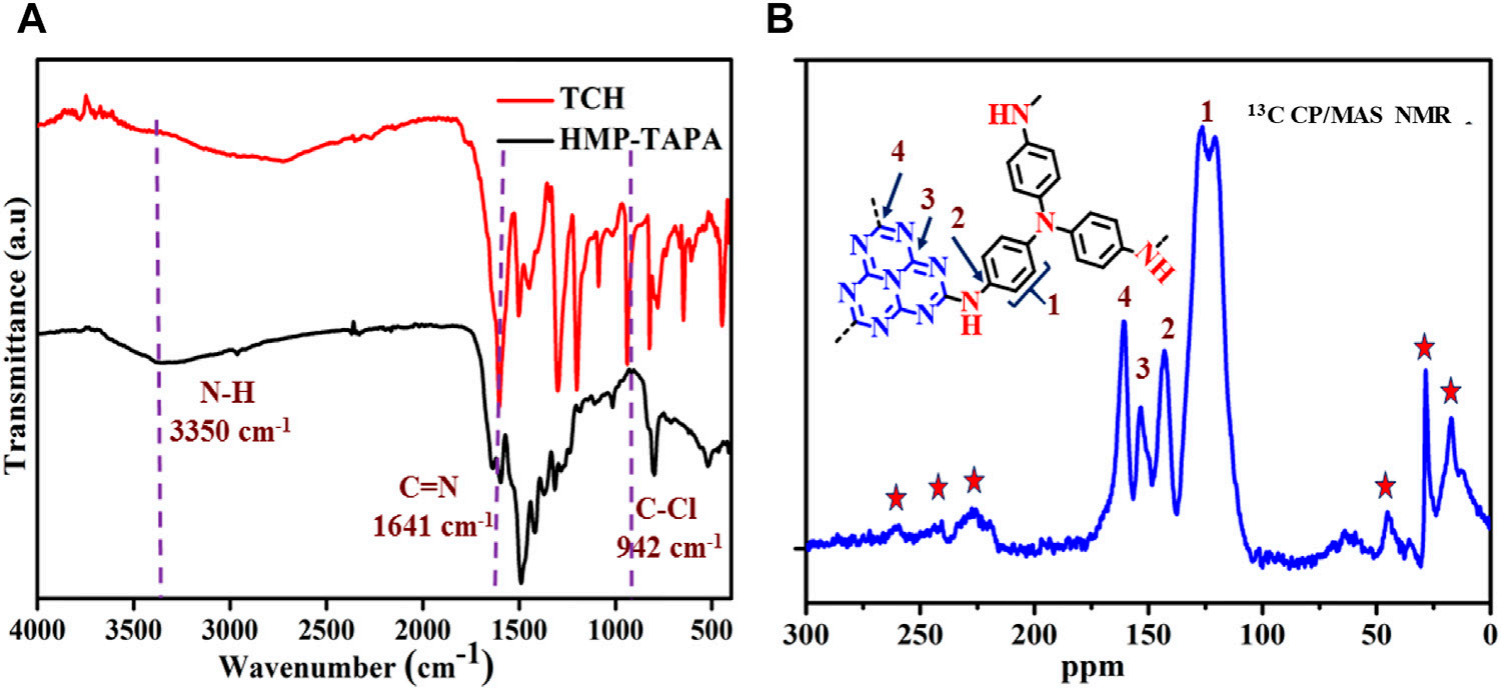
FTIR **(A)** and ^13^C CP/MAS NMR **(B)** spectra of HMP-TAPA represent the spinning side bands.

In order to explicate the structure of HMP-TAPA, FTIR and, ^13^C CP/MAS, NMR spectroscopy was performed. As shown in Figure 1A, FTIR spectra of HMP-TAPA display an intense peak at 800 cm^−1^, which corresponds to the breathing mode of the heptazine moiety. The absorption band at 1641 cm^−1^ harmonized to the C=N stretching vibration of the heptazine moiety, which could be further validated by vanishing of C-Cl stretching vibration at 942 cm^−1^ in HMP-TAPA (Ma et al., 2016).

Additionally, ^13^C CP/MAS NMR spectroscopy also confirmed the structure of HMP-TAPA. In Figure 1B, HMP-TAPA exhibits an NMR signal at 162 and 156 ppm corresponding to the sp^2^-hybridized carbon of the heptazine moiety. Furthermore, the disappearance of the NMR peak at 176 ppm suggests the complete substitution of three chlorine atoms. The NMR signal in the range of 110–150 ppm corresponds to the sp^2^ carbon of the TAPA precursor. Furthermore, the PXRD pattern reveals the amorphous nature of the material (Supplementary Figure S2). This might be due to the irreversible kinetic control during the polymerization process of HMP-TAPA, which resulted in the disorderly growth of the framework (Ma et al., 2016).

### Gas Adsorption Properties

**FIGURE 2:**
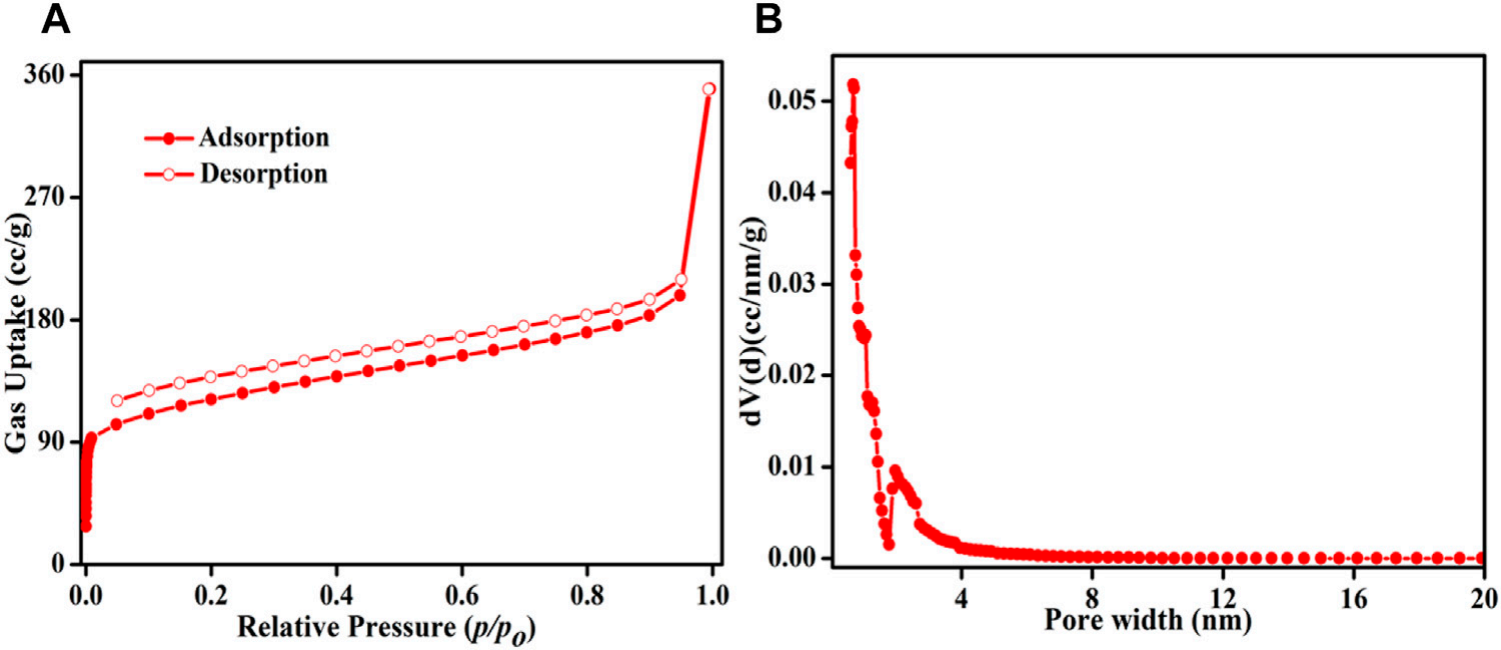
**(A)** N_2_ adsorption–desorption isotherms carried out at 77 K for HMP-TAPA. Closed circles represent adsorption and open circles for desorption. **(B)** Pore size distribution in HMP-TAPA.

After probing the chemical structure of the framework, the porous nature of the catalyst was investigated by measuring N_2_ adsorption–desorption isotherms at 77 K (Figure 2A). HMP-TAPA shows type-I isotherm indicating the presence of a micropore region with precipitous nitrogen uptake at the low-pressure region. The hysteresis loop indicates the presence of mesopores in HMP-TAPA. The Brunauer−Emmett−Teller (BET) surface area was calculated to be 424 m^2^/g. The majority of pores lies in the region of 0.7–1.2 nm, and some of the pores are of 2–4 nm, as suggested by pore size distribution (PSD) obtained by using the NLDFT method from the desorption branch of the N_2_ isotherm (Figure 2B). In Figure 2A, the observed hysteresis loop is not closed but open. It could also be explained by the swelling effects and softness of the porous organic polymeric network (Weber et al., 2008).

The nitrogen (N) content of HMP-TAPA was estimated around 27.81% (C, H, and residue) by CHN analysis. This high N content, which also corresponds to the Lewis basic nature of framework, may be considered to increase its efficiency toward CO_2_ sorption and also its selectivity for CO_2_ molecules. It has been well-documented that N-rich frameworks with increased Lewis basic sites render high selectivity.

**FIGURE 3:**
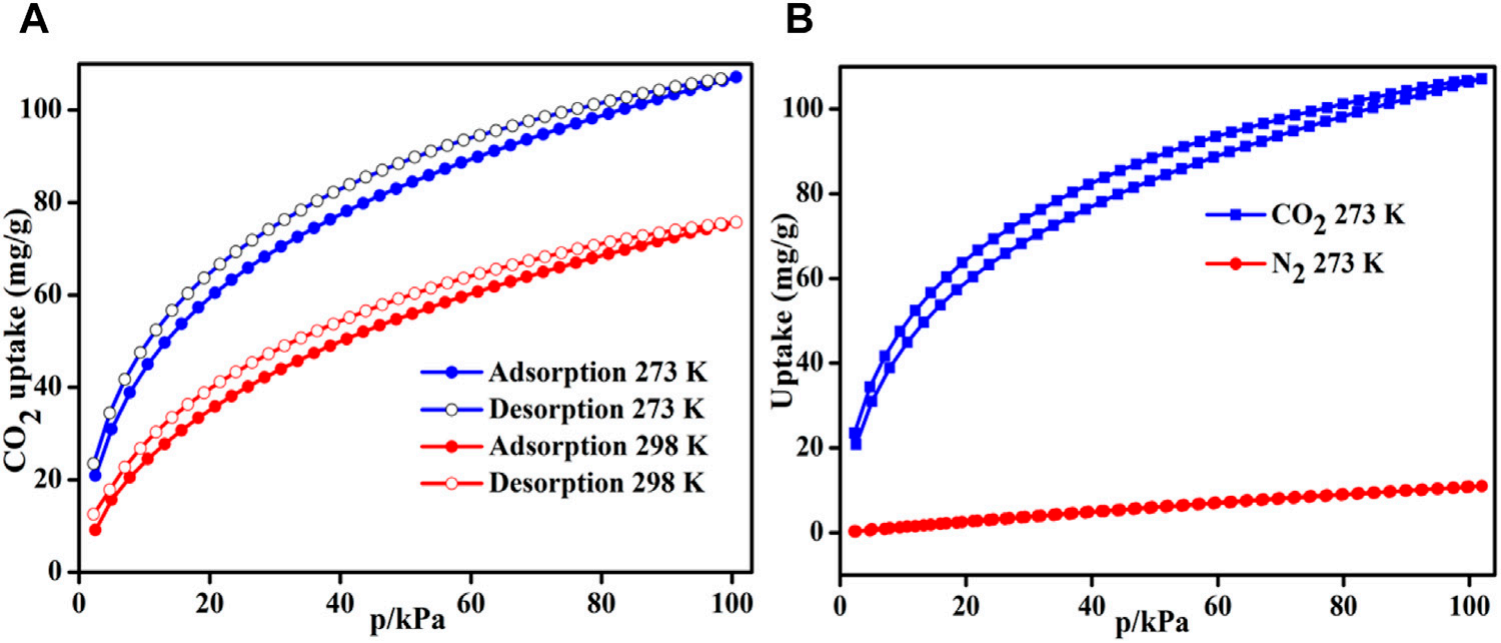
**(A)** CO_2_ uptake at 273 and 298 K. **(B)** CO_2_ and N_2_ adsorption isotherm of HMP-TAPA at 273 K.

The selectivity for CO_2_ over N_2_ is an important requirement for CO_2_ adsorbence since the burning of fossils emits fuel gases that contain 15–16% CO_2_ having 0.15 bar partial pressure^.^ Thus, to investigate the separation behavior of heptazine-based porous polymers, binary mixture selectivity at two different temperatures was estimated by the IAST method developed by Myers and Prausnitz (Myers and Prausnitz, 1965). It shows higher CO_2_ uptake than N_2_, which is about 106.7 mg/g and 11 mg/g at 273 K, (Figure 3B), respectively, (1 bar), and thus, it shows high selectivity of the heptazine-based framework toward CO_2_ molecules than N_2_. Furthermore, the Langmuir–Freundlich isotherm model was used for fitting the isolated sorption isotherms for CO_2_ and N_2_ (Supplementary Figures S3–S5). The observed selectivity for HMP-TAPA is 26.27 and 30.97 at 273 and 298 K, respectively, which is the highest achieved value among the heptazine-based porous polymers reported so far (Dang et al., 2015). Moreover, the strong affinity of heptazine-based porous frameworks toward CO_2_ has been explained on the basis of the dipole–quadrupole interaction between the N atoms in the pores and the adsorbed CO_2_ molecules (Oschatz and Antonietti, 2018). Thus, the selective CO_2_ sorption could be easily attributed to a high surface area and rich N content of the porous framework.

**FIGURE 4:**
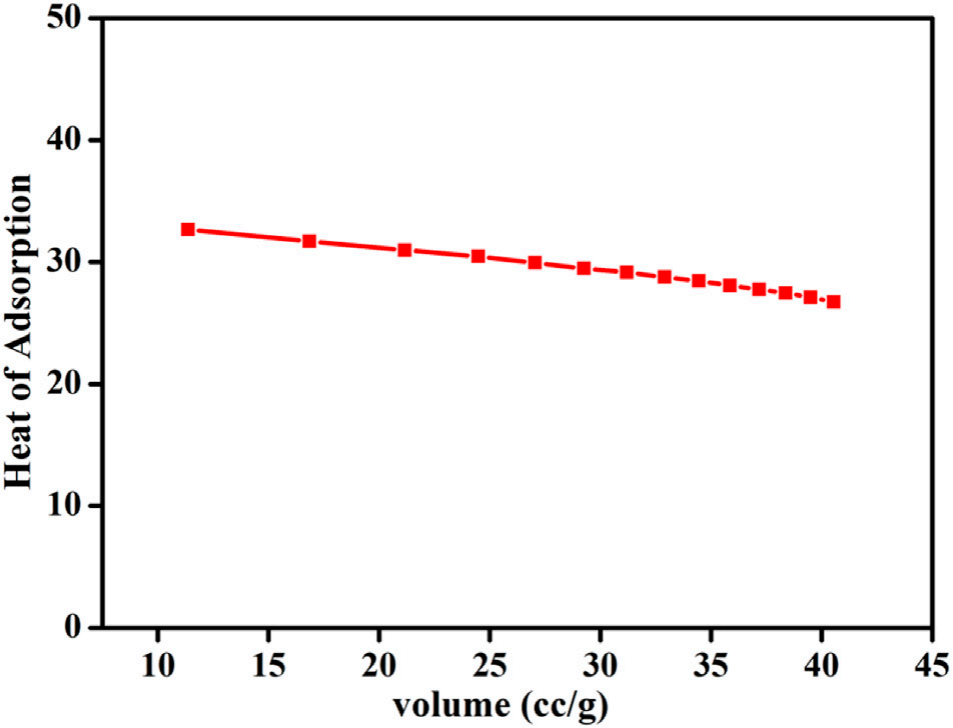
Isosteric heat of the adsorption curve of HMP-TAPA for CO_2_.

To gain further insights into CO_2_ adsorption efficiency, isosteric heat of adsorption (Q_st_) was calculated from the CO_2_ adsorption isotherm at 273, 281, and 298 K, according to the Clausius–Clapeyron equation (Figure 4). The Q_st_ value measures the initial interaction strength at low gas loading and also establishes the physisorption/chemisorption interaction nature between the adsorbent gas (CO_2_) and the porous framework (HMP-TAPA). The Q_st_ value was calculated to be 32.8 kJ/mol, which suggested a moderate interaction between CO_2_ molecules and free -N-, N–H, and -NH_2_ groups over the surface of the HMP-TAPA polymeric network.

### Chemical and Thermal Stability of HMP-TAPA

**FIGURE 5:**
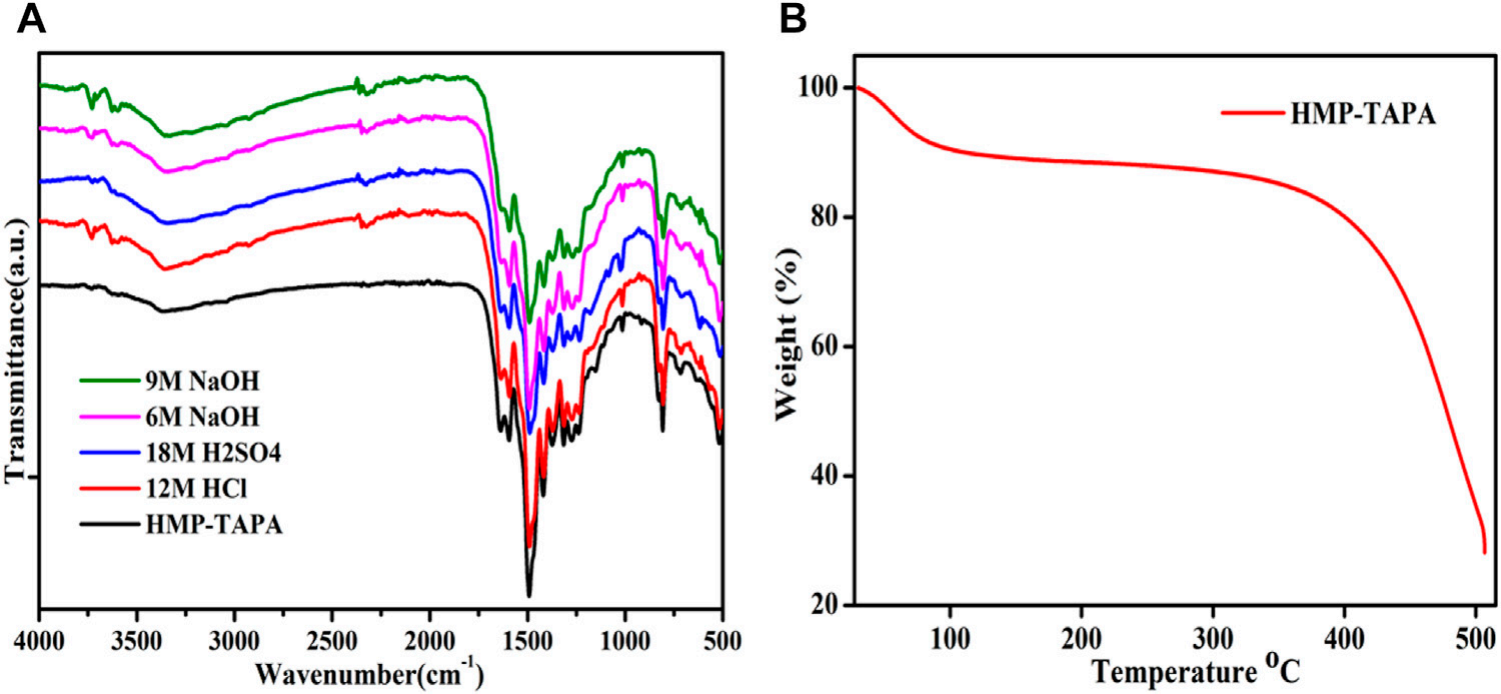
**(A)** Stability studies for HMP-TAPA analyzed using FTIR spectra over a period of 7 days in different solvents. **(B)** TGA plot for HMP-TAPA.

Interestingly, the as-synthesized porous organic polymer (HMP-TAPA) was found to exhibit high chemical and thermal stability. To test the chemical stability, HMP-TAPA was soaked in different organic solvents (ranging from highly protic to aprotic) with vigorous stirring for 7 days and then the solids were recovered by filtration and washed thoroughly. Water stability was tested at various pH conditions in which HMP-TAPA was stirred in 6 and 9 M NaOH, 18 M H_2_SO_4_, and 12 M HCl solutions for 10 days (Figure 5A). HMP-TAPA was also found to be insoluble in boiling water, and this proved the remarkable stability of HMP-TAPA, as shown using FTIR (Figure 5A). This unusual behavior could be originating due to the heptazine core of the framework and the close stacking of aromatic rings, making the surface more hydrophobic.

The thermal stability of the synthesized HMP-TAPA was determined by TGA. The TGA profile showed stability of HMP-TAPA up to 420°C, suggesting high thermal stability. The weight loss between 30°C and 100°C could be assigned to the removal of any organic solvent/moisture present in the framework (Figure 5B).

### Catalytic Cycloaddition of Carbon Dioxide With Epoxides

**SCHEME 2:**
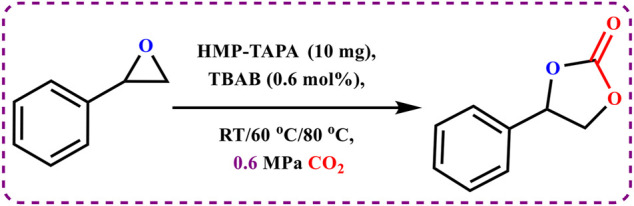
Cycloaddition of CO_2_ with epoxides catalyzed by HMP-TAPA.

A temperature-programmed desorption (TPD) measurement was performed to detect the concentration of basic sites under gaseous CO_2_ flow. TPD analysis showed two peaks at 92°C and 352°C, corresponding to the presence of weak and strong basic sites, respectively, as shown in Supplementary Figure S6. Mainly, the large number of N–H groups on the surface of HMP-TAPA accounted for the strong basic sites. However, the entire concentration of the basic sites was estimated to be 278 μmol/g. Motivated by the high thermal stability and chemical stability along with selective CO_2_ capture properties and high basic nature of HMP-TAPA, we envisioned that it can act as an efficient heterogeneous catalyst for the cycloaddition of CO_2,_ as shown in Scheme 2. Therefore, the catalytic activity of HMP-TAPA was investigated for the cycloaddition reaction using styrene oxide as a model substrate (epoxide) at different conditions of CO_2_ pressure and tetra-butyl ammonium bromide (TBAB) as the co-catalyst.

To our surprise, the catalytic results showed the formation of the corresponding cyclic carbonate with about 98% conversion in 12 h (Table 1, entry nos. 1-9). Furthermore, controlled experiments revealed that both HMP-TAPA and TBAB are essential for the catalytic activity (Table 1, entry nos. 1–9). It is known that TBAB acts as a nucleophilic co-catalyst and facilitates the ring opening of the epoxides.

**TABLE 1:** Optimization of reaction conditions for cycloaddition of CO_2_ with styrene oxide catalyzed by HMP-TAPA.

Entry No	Catalyst [mol%]	Co-catalyst [mol%]	Pressure [MPa]	Time [h]	Temp. [°C]	Conversion [%]^a^
1	None	None	0.1	12	r.t	NIL
2	None	None	0.6	12	r.t	NIL
3	None	None	0.1	12	80	NIL
4	None	None	0.6	12	80	NIL
5	HMP-TAPA	None	0.4	12	80	36
6	None	TBAB	0.4	12	80	34
7	TCH & TAPA^b^	TBAB	0.4	12	80	56
8	HMP-TAPA	TBAB	0.4	12	80	83
9	HMP-TAPA	TBAB	0.6	12	80	98

Reaction conditions: Styrene oxide (10 mmol), 0.1–0.6 MPa CO_2_ pressure, Catalyst:TBAB (0.1mol%).

a The catalytic conversions were determined by ^1^H NMR analysis using 1, 1’, 2, 2’-tetrachloroethane as an internal standard.

b Physical mixture of starting precursor used instead of using POP.

**FIGURE 6:**
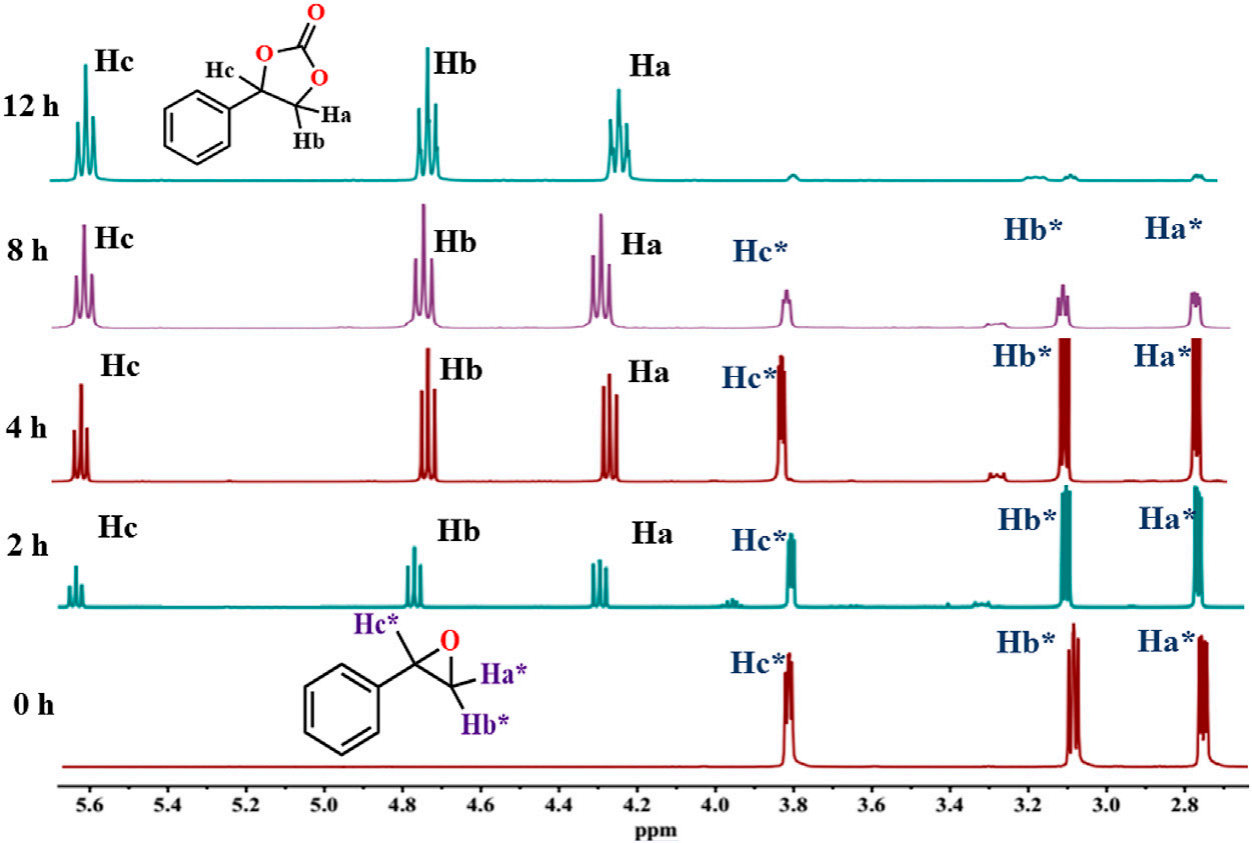
Time-dependent ^1^H CDCl_3_, 400 MHz NMR stack plot for the cycloaddition of CO_2_ with styrene oxide catalyzed by HMP-TAPA showing gradual increase of peaks at 4.3, 4.8, and 5.6 ppm corresponds to the product, and decrease in the intensity of peaks around 2.7, 3.1, and 3.8 ppm corresponds to styrene oxide.

With no additional by-products, 100% selectivity for cyclic carbonates was obtained (Figure 6). The progress of the catalytic reaction can be easily monitored by ^1^H NMR spectra of the aliquots taken at regular time intervals with reference to an internal standard (1,1′,2,2′-tetra-chloroethane). The time-dependent ^1^H NMR of styrene oxide is been shown in Figure 6.

The optimum conditions required for the cycloaddition reaction were investigated from the controlled experiments which revealed that a temperature of 80°C with catalyst:TBAB loading of (0.1 mol%) and CO_2_ pressure of 0.6 MPa are needed (Table 1, Entry no. 9). Having confirmed the optimum conditions for HMP-TAPA, the catalytic activity was further extended to other epoxides having different lengths of alkyl and aromatic units. Interestingly, the catalytic conversion of alkyl epoxides such as 1, 2-epoxybutane, 1, 2-epoxyhexane, and 1, 2-epoxydecane, were found to be lower than that of 1,2-epoxypropane, 2-methyl-1,2-epoxy propane, and epichlorohydrin, which can be attributed to their reduced activity owing to the presence of an alkyl chain which is shorter in case of the first three compounds (Table 2, entries 1 and 3 and Supplementary Figures S7–S8). Thus, the catalytic conversion can be correlated to the confinement in the pores of HMP-TAPA and restricted diffusion of the compounds with larger chain lengths having much slower activity (Table 2, entry 4 to 6 and Supplementary Figures S8–S10). The higher catalytic activity of HMP-TAPA can be attributed to the presence of high density of basic nitrogen sites, resulting in enhanced activity for selective CO_2_ capture and conversion. Also, butyl glycidyl ether gives relatively higher catalytic conversion over allyl glycidyl ether which can be ascribed to the electron-donating nature of the former than the latter (Table 2, entry 7-8 and Supplementary Figures S11–S12). Furthermore, aromatic epoxides, such as styrene oxide and phenyl glycidyl ether, were investigated for cycloaddition reaction, which showed a conversion of 81 and 57%, respectively. With the extended time for aromatic epoxides, it was found that styrene oxide takes 12 h and the latter takes 15 h for the complete conversion. (Table 2, entries 9–10 and Supplementary Figures S13–S14). It should be noted that the reaction conditions used in this study for the cycloaddition reaction are relatively milder than the conditions normally employed in the literature for triazine-based frameworks, as shown in Supplementary Table S1.

**TABLE 2:** Cycloaddition reaction of various substituted epoxides catalyzed by HMP-TAPA.
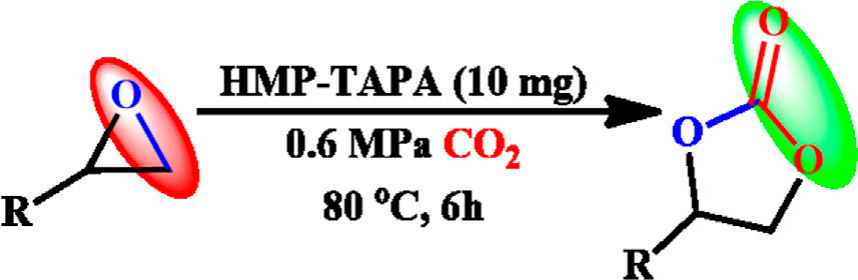

	
Entry No	Substrate [R]	Pressure^a^ [MPa]	Conversion^b^ [%]	TON^c^	TOF^d^
1	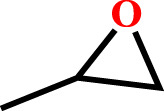	0.6	>99	472	78.7
2	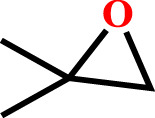	0.6	>98	467	77.8
3	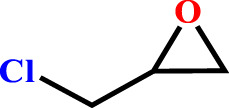	0.6	>99	472	78.7
4	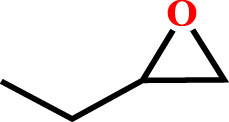	0.6	97	462	77
5	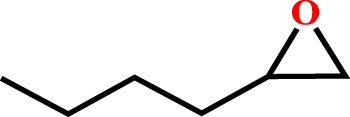	0.6	81	386	64.3
6	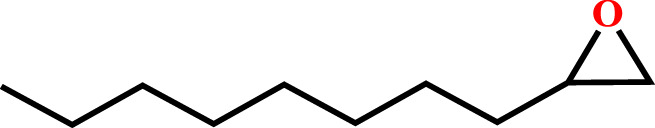	0.6	61	291	48.4
7	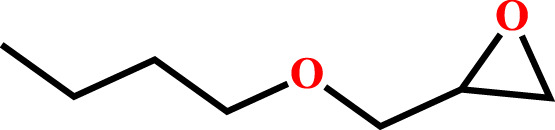	0.6	71	338	56.3
8	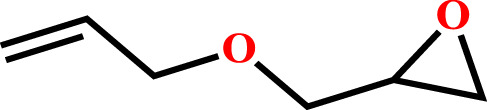	0.6	67	319	53.1
9	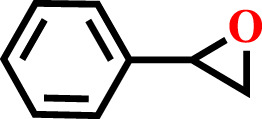	0.6	81/98^e^	386/467	64.3
10	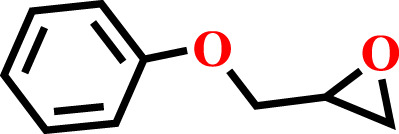	0.6	57/98^f^	271/467	45.1

a Reaction conditions: epoxide (10 mmol), HMP-TAPA catalyst (10 mg), temperature 80°C, and time 6 h

b Conversion: the catalytic conversions were determined by ^1^H NMR analysis.

c TON: moles of cyclic carbonate/mol of catalyst used (considering the monomers used).

d TOF: TON/time of reaction in hours.

e Reaction time 12 h.

f Reaction time 15 h.

**FIGURE 7:**
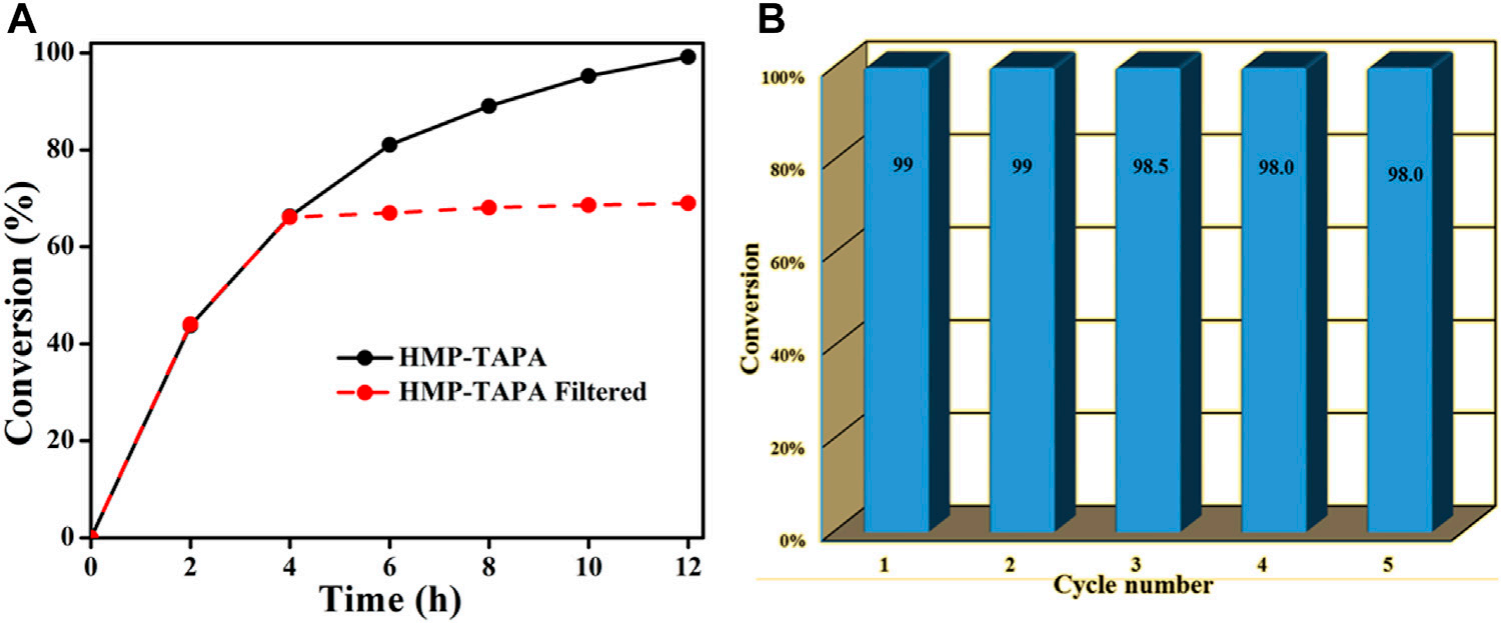
**(A)** Leaching test for the HMP-TAPA catalyst removed after 4 h. **(B)** Recycling test of HMP-TAPA for five successive cycles.

To rule out the possibility of homogeneous catalysis by HMP-TAPA precursors or monomers leached into the solution, the reaction was stopped at 4 h, and the conversion of styrene oxide was found to be ∼63%. Then the HMP-TAPA catalyst was removed by filtration, and the filtrate was allowed to stir for an additional 8 h in the presence of 6 bar pressure of CO_2_. (Figure 7A). The analysis of the aliquot taken at 12 h revealed a slight increase (∼2%) in the conversion of styrene oxide, which is considerably lower than the reaction carried out in the presence of HMP-TAPA. Furthermore, HMP-TAPA was easily separated from the reaction mixture by simple filtration and reused for subsequent cycles after washing with DCM/MeOH. Remarkably, the catalytic activity of HMP-TAPA was retained even after five consecutive cycles (Figure 7B). Also, the FT-IR spectra of the recovered sample were recorded, and it was found that the structure integrity and functional groups present were intact after catalysis (Supplementary Figure S15).

### Plausible Mechanism

**SCHEME 3:**
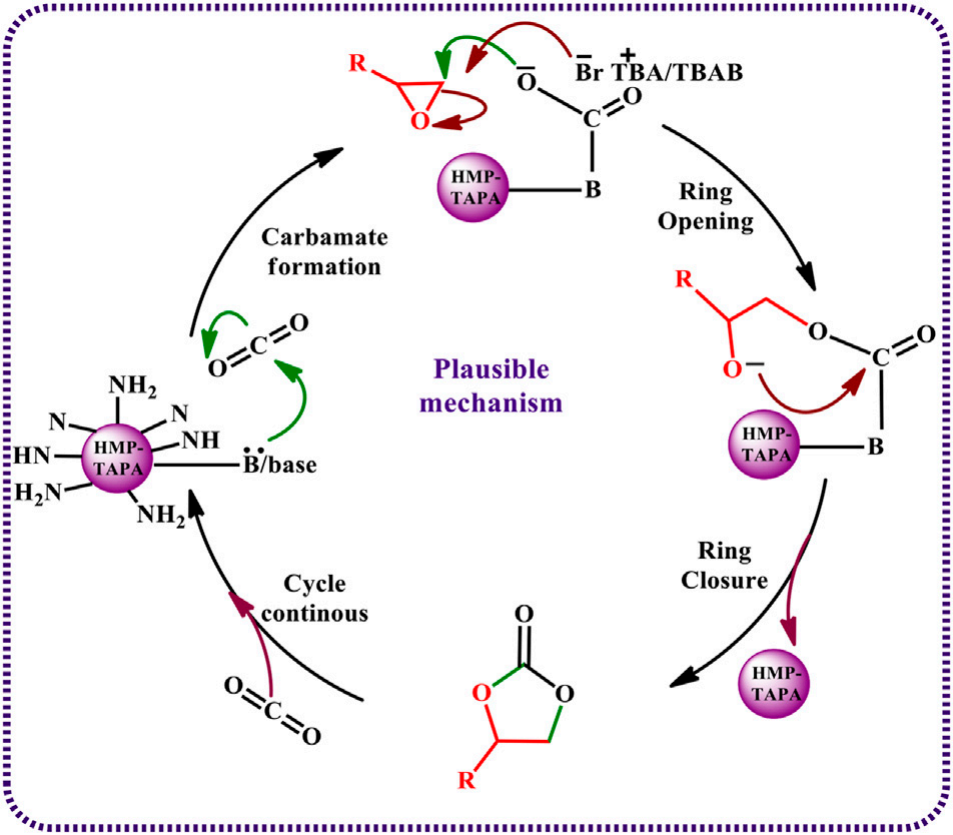
Proposed reaction mechanism for cycloaddition of CO_2_ with epoxides catalyzed by HMP-TAPA.

The proposed mechanism for the HMP-TAPA–catalyzed cycloaddition of CO_2_ with epoxides is shown in Scheme 3. The first step involves the polarization and subsequent coordination of CO_2_ at the basic -N-, -NH-, and -NH_2_- moieties present in TAPA and heptazine of HMP-TAPA, resulting in the formation of carbamate species which acts as a nucleophile. Furthermore, these carbamate nucleophiles attack epoxides present on the surface and assist in the ring opening of the epoxide. Also, TBAB helps in faster ring opening of epoxides, which further facilitates the selectivity of the cyclic carbonate in the reaction. The subsequent ring closure reaction by an intramolecular nucleophilic attack of the oxyanion with CO_2_ leads to the formation of cyclic carbonate. Then the reductive elimination of the cyclic carbonate regenerates the catalyst for incoming molecules of CO_2_ and epoxides, making the catalytic cycle as a continuous process.

## Conclusion

The microporous polymeric network (HMP-TAPA) composed of a heptazine core shows the highest surface area among these classes of polymeric networks. The presence of a large number of -N-, -NH, and -NH_2_ groups on the surface of HMP-TAPA remarkably enhances the basicity of the polymer, which synergistically improved the CO_2_ sorption. HMP-TAPA exhibits permanent porosity with an IAST selectivity of 30.97 at 273 K ,which is highest among the heptazine-based porous polymers reported so far. In addition, the catalytic activity of HMP-TAPA was scrutinized for efficient and recyclable catalysts for the cycloaddition of CO_2_ with epoxides at relatively mild conditions, 0.6 MPa of CO_2_ and 80°C to generate cyclic carbonates in high yield and selectivity. The HMP-TAPA catalyst can be recycled for five successive cycles without significant loss of catalytic activity. The current study thus provides a hint that the heptazine-based porous organic polymers (HMPs) could be utilized for a wide variety of organic conversions (both thermal and photo-catalysis) and expecting more reports in the near future.

### Supporting Information

PXRD plots of the compounds, FTIR, additional figures, gas adsorption–desorption isotherms, ^1^H NMR spectra, ^13^C NMR of heptazine chloride, CO_2_ TPD data, IAST selectivity, Langmuir–Freundlich, reaction in different solvents, and IR data to check chemical stability.

## Data Availability

The original contributions presented in the study are included in the article/Supplementary Material; further inquiries can be directed to the corresponding author.
