# Supplementary material for: Metal-Free Heptazine-Based Porous Polymeric Network as Highly Efficient Catalyst for CO2 Capture and Conversion
Source: Front Chem. 2021 Oct 15;9:737511. doi: 10.3389/fchem.2021.737511 (PMC8554583; doi:10.3389/fchem.2021.737511)
Supplement: Supplementary file 1 [file Data_Sheet_1.docx]

**Supporting Information**

**Metal-Free Heptazine Based Porous Polymeric Network as Highly Efficient Catalyst for CO_2_ Capture and Conversion**

Neha Sharma, Bharat Ugale, Sunil Kumar and Kamalakannan Kailasam*

Advanced Functional Nanomaterials, Energy and Environment Unit, Institute of Nano Science and Technology (INST), Knowledge city, Sector-81, Manauli, SAS Nagar, 140306 Mohali , Punjab, India.

Email: [kamal@inst.ac.in](mailto:kamal@inst.ac.in)

**Contents:**

1. **Material and monomer synthesis**
2. **Figure S1.** ^13^C NMR of Heptazine chloride.
3. **Figure** **S2**. PXRD plot of HMP-TAPA.
4. **Figure S3.** Nitrogen isotherm at 273 K (symbols) and Langmuir-Freundlich equation fit (line) for HMP-TAPA.
5. **Figure S4**. Carbon dioxide isotherm at 273 K (symbols) and Langmuir-Freundlich equation fits (line) for HMP-TAPA.
6. **Figure S5.** TPD Data of HMP-TAPA.
7. **Figure S6.** ^1^H NMR (CDCl_3_, 400 MHz) spectra for the cycloaddition reaction of propylene oxide with CO_2_ catalyzed by HMP-TAPA (Table 2, entry no. 1).
8. **Figure S7.** ^1^H NMR (CDCl_3_, 400 MHz) spectra for the cycloaddition reaction of epichlorohydrin with CO_2_ using HMP-TAPA as catalyst (Table 2, entry no. 3).
9. **Figure S8.** ^1^H NMR (CDCl_3_, 400 MHz) spectra for the cycloaddition reaction of butylene oxide with CO_2_ catalyzed by HMP-TAPA (Table 2, entry no. 4).
10. **Figure S9.** ^1^H NMR (CDCl_3_, 400 MHz) spectra for the cycloaddition reaction of 1,2-epoxyhexane with CO_2_ catalyzed by HMP-TAPA (Table 2, entry no. 5).
11. **Figure S10.** ^1^H NMR (CDCl_3_, 400 MHz) spectra for the cycloaddition reaction of 1,2-epoxydecane with CO_2_ catalyzed by HMP-TAPA (Table 2, entry no. 6).
12. **Figure S11.** ^1^H NMR (CDCl_3_, 400 MHz) spectra for the cycloaddition reaction of butyl glycidyl ether with CO_2_ catalyzed by HMP-TAPA (Table 2, entry no. 7).
13. **Figure S12.** ^1^H NMR (CDCl_3_, 400 MHz) spectra for the cycloaddition reaction of allyl glycidyl ether with CO_2_ catalyzed by HMP-TAPA (Table 2, entry no. 8).
14. **Figure S13.** ^1^H NMR (CDCl_3_, 400 MHz) spectra for the cycloaddition reaction of styrene oxide with CO_2_ catalyzed by HMP-TAPA (Table 2, entry no. 9).
15. **Figure S14.** ^1^H NMR (CDCl_3_, 400 MHz) spectra for the cycloaddition reaction of phenyl glycidyl ether with CO_2_ catalyzed by HMP-TAPA (Table 2, entry no. 10).
16. **Figure S15.** FT-IR spectra for HMP-TAPA and the recovered sample after five catalytic cycles.
17. **Table S1**. Comparison of the reaction conditions used for cycloaddition of CO_2_ by various triazine based framework by taking epichlorohydrin as model substrate.

**Material and monomer synthesis**

**
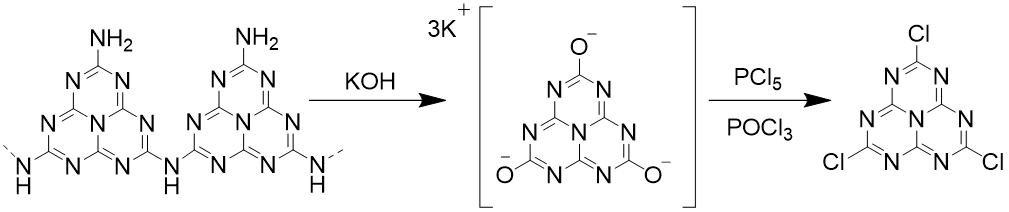
**

**Yield = ~ 40%**


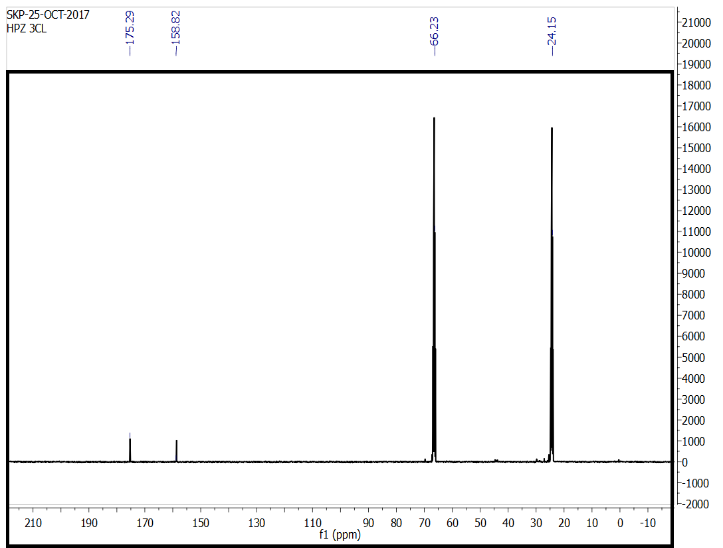


**Figure S1.** ^13^C NMR of Heptazine chloride.

**Figure** **S2**. PXRD plot of **HMP-TAPA**.

**Analysis of gas adsorption isotherms**

**IAST Selectivity:**

Gas selectivity of mixture at different temperatures were calculated based on Ideal Adsorbed Solution Theory (IAST) proposed by Mayer and Praunitz. In order to calculate the sorption selectivity of CO_2_ and N_2_ mixture using HMP-TAPA the values used were fitted from the single component of CO_2_ and N_2_ adsorption based on Langmuir –Freundlich model and parameter used are given as follows

Y=B*x^(1/t)/[1+B*x^(1/t)]*Q

The predicted adsorption selectivity is defined

S_=_$\frac{\frac{x1}{y1}}{\frac{x2}{y2}}$

Where,

xi and yi are the mole fraction of component in the adsorbed and bulk phase. The IAST calculation was carried out for binary reaction mixture containing 15% CO_2_ (y1) and 85% N_2_  (y2).





**Figure S3.** Nitrogen isotherm at 273 K (symbol) and Langmuir-Freundlich equation fit (line) for **HMP-TAPA**.





**Figure S4.** Carbon dioxide isotherm at 273 K (symbol) and Langmuir-Freundlich equation fit (line) for **HMP-TAPA**.





**Figure S5.** TPD Data of **HMP-TAPA**.

**
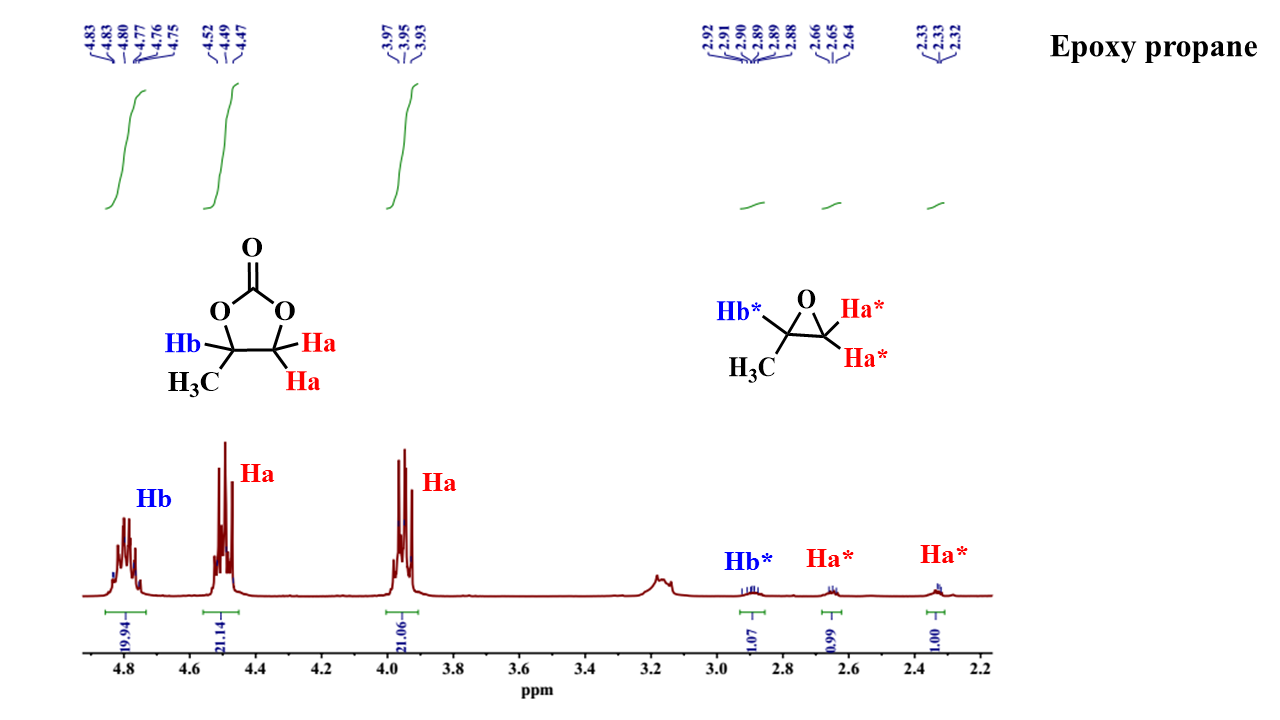
**

**Figure S6.** ^1^H NMR (CDCl_3_, 400 MHz) spectra for the cycloaddition reaction of propylene oxide with CO_2_ catalyzed by **HMP-TAPA** (Table 2, entry no. 1).


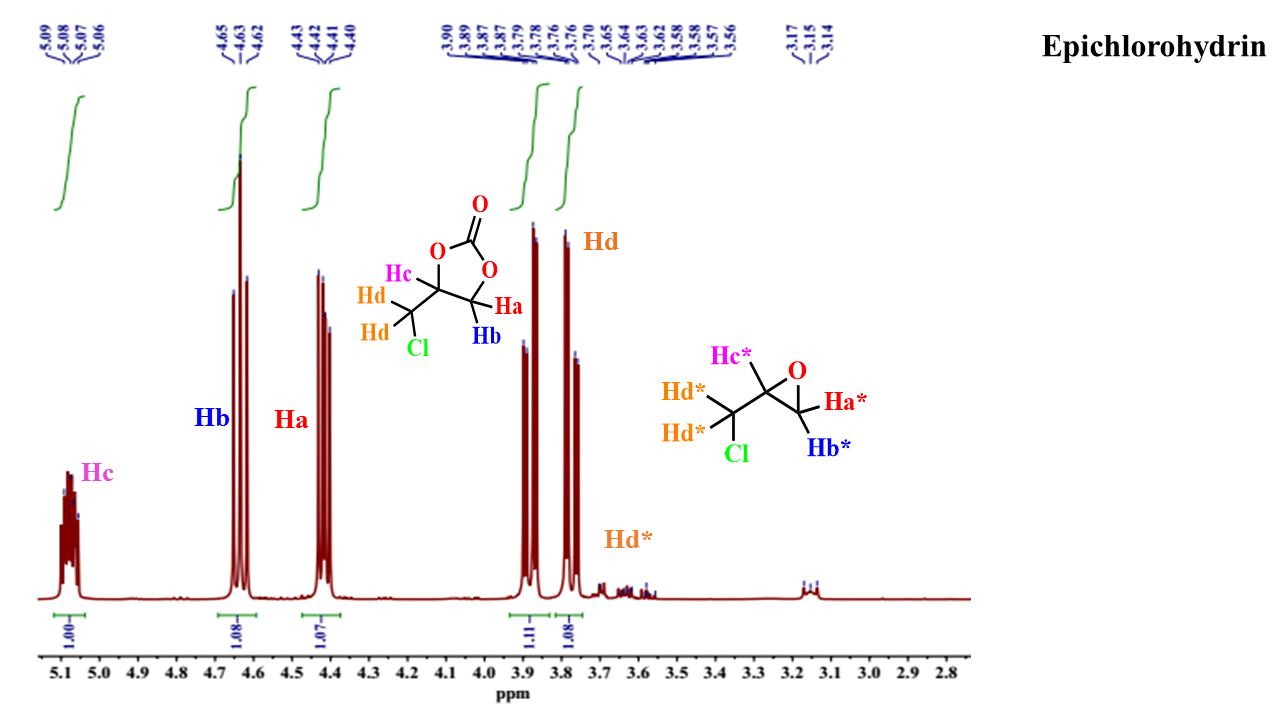


**Figure S7.** ^1^H NMR (CDCl_3_, 400 MHz) spectra for the cycloaddition reaction of epichlorohydrin with CO_2_ using **HMP-TAPA** as catalyst (Table S4, entry no. 3).


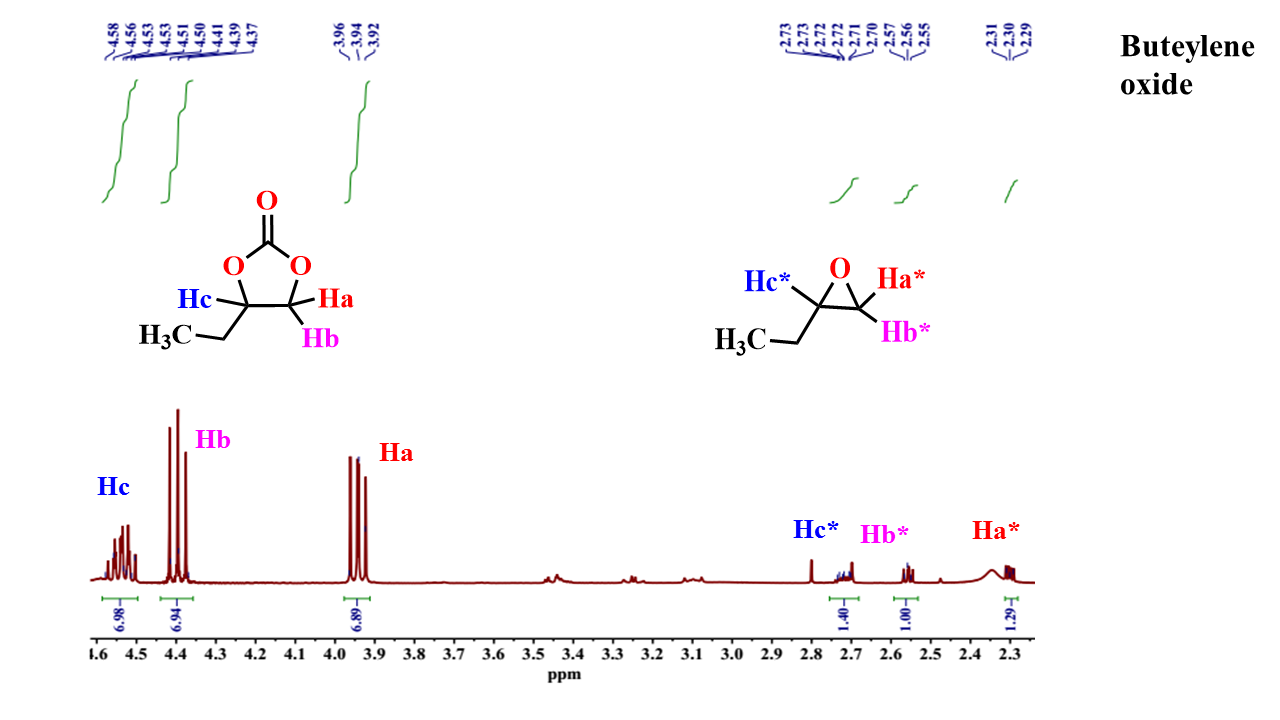


**Figure S8.** ^1^H NMR (CDCl_3_, 400 MHz) spectra for the cycloaddition reaction of butylene oxide with CO_2_ catalyzed by **HMP-TAPA** (Table 2, entry no. 4).


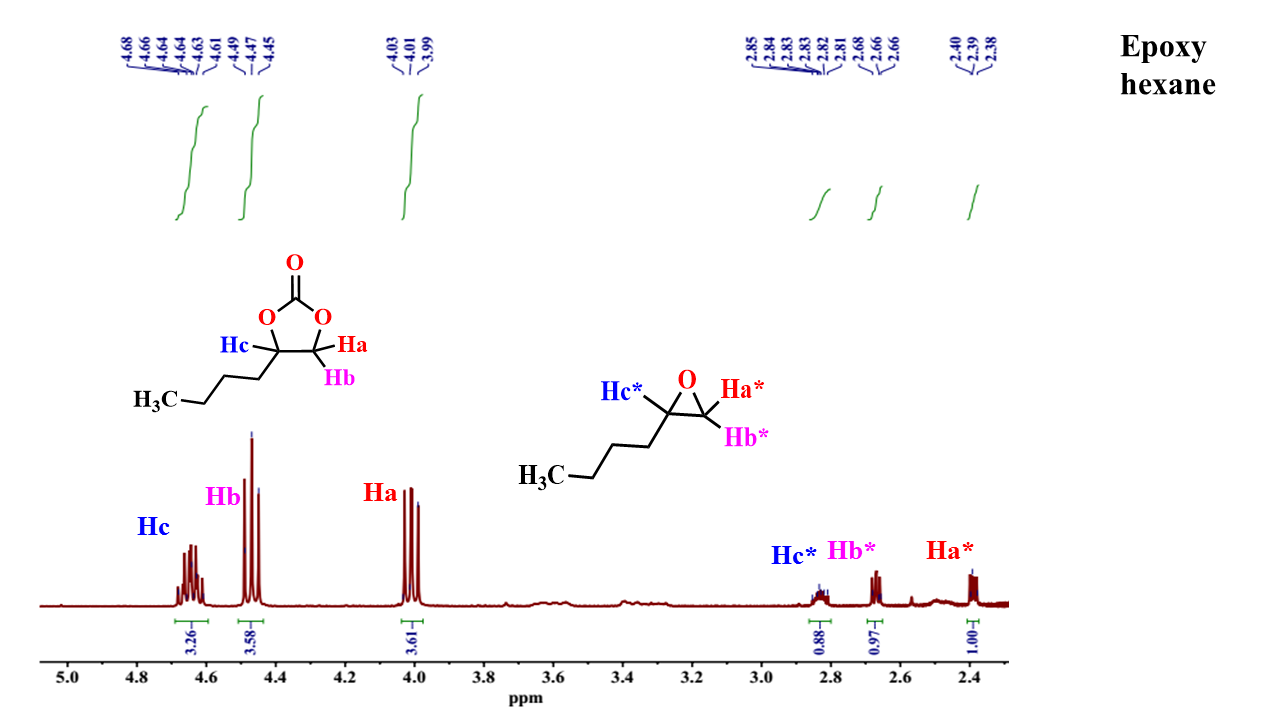


**Figure S9.** ^1^H NMR (CDCl_3_, 400 MHz) spectra for the cycloaddition reaction of 1,2-epoxyhexane with CO_2_ catalyzed by **HMP-TAPA** (Table 2, entry no. 5).


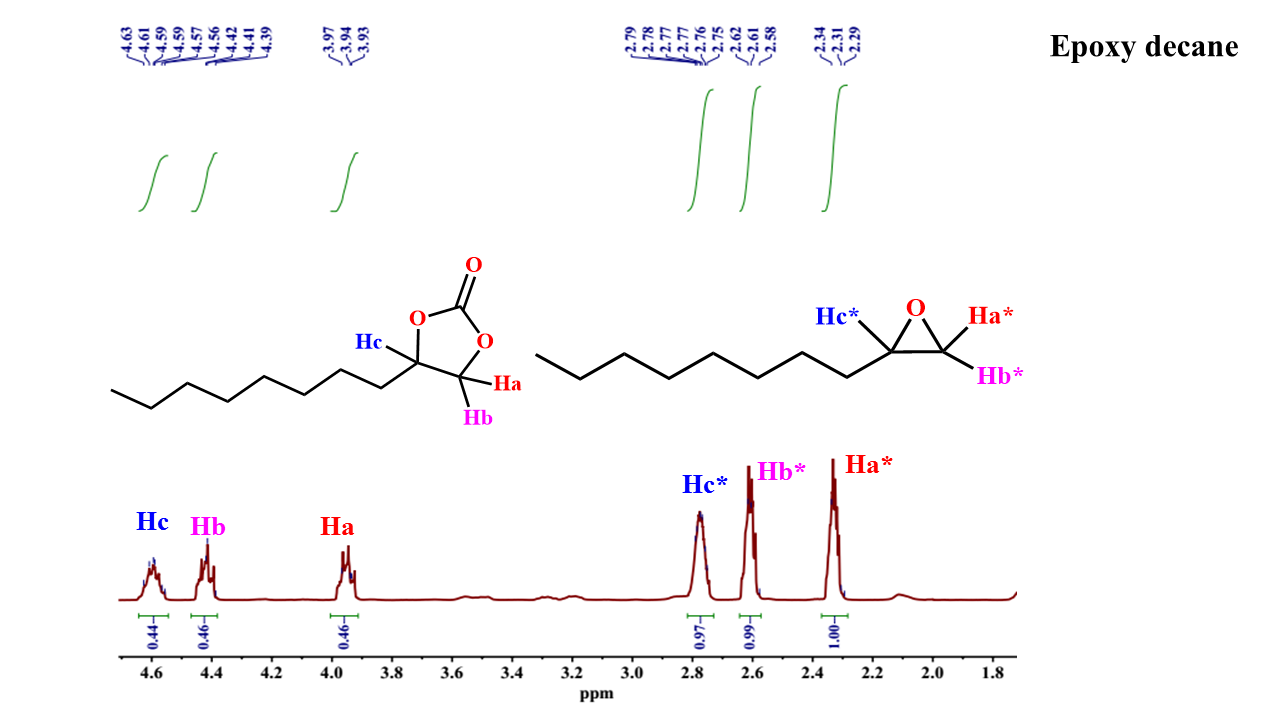


**Figure S10.** ^1^H NMR (CDCl_3_, 400 MHz) spectra for the cycloaddition reaction of 1,2-epoxydecane with CO_2_ catalyzed by **HMP-TAPA** (Table 2, entry no. 6).

**
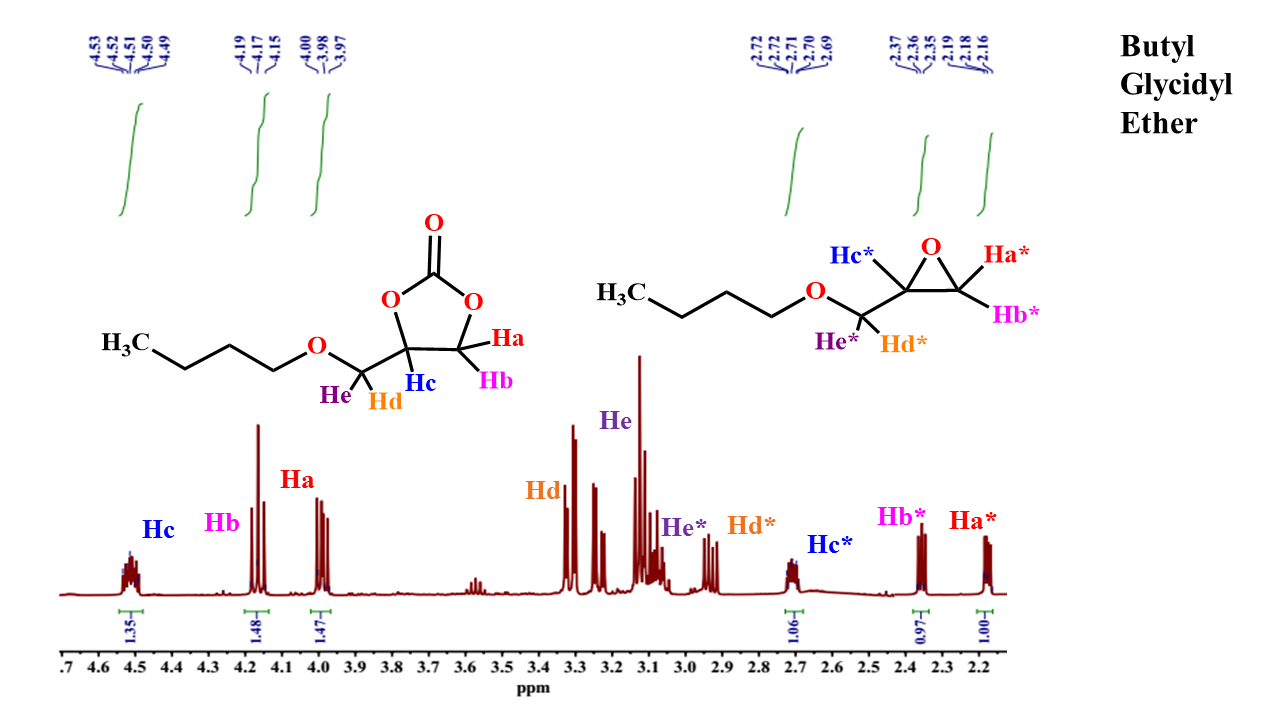
**

**Figure S11.** ^1^H NMR (CDCl_3_, 400 MHz) spectra for the cycloaddition reaction of butyl glycidyl ether with CO_2_ catalyzed by **HMP-TAPA** (Table 2, entry no. 7).

**
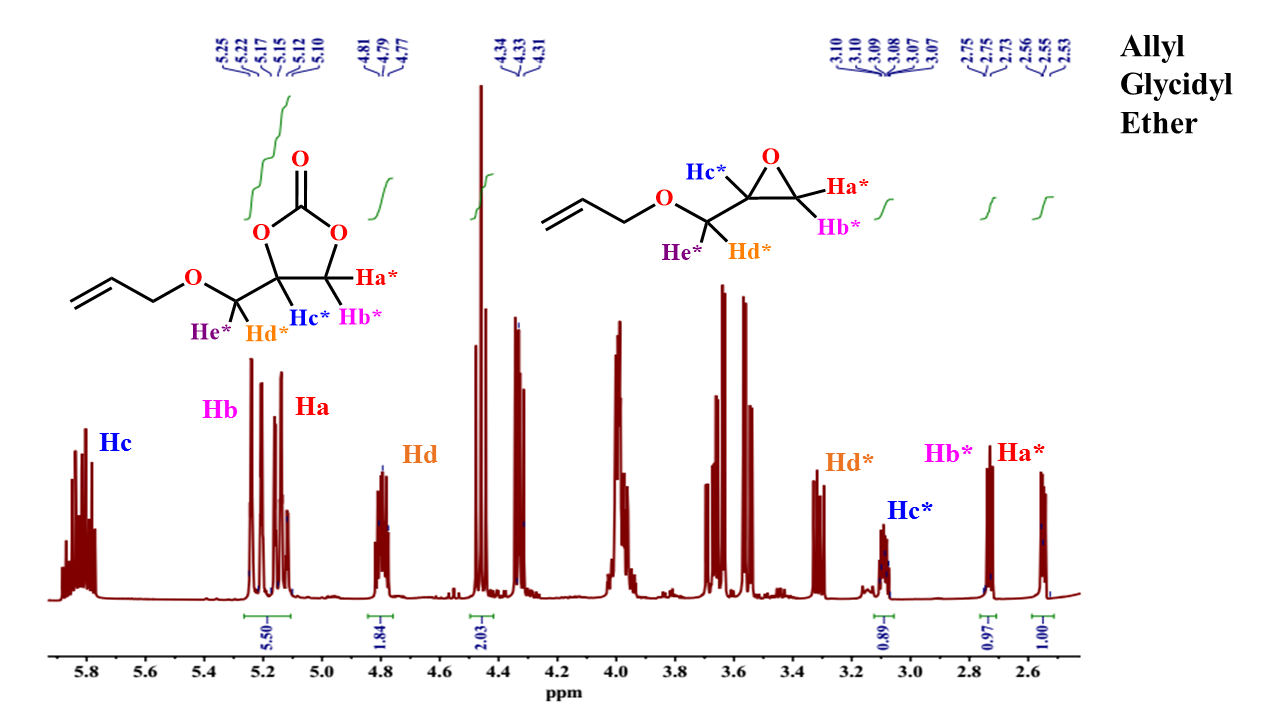
**

**Figure S12.** ^1^H NMR (CDCl_3_, 400 MHz) spectra for the cycloaddition reaction of allyl glycidyl ether with CO_2_ catalyzed by **HMP-TAPA** (Table 2, entry no. 8).

**
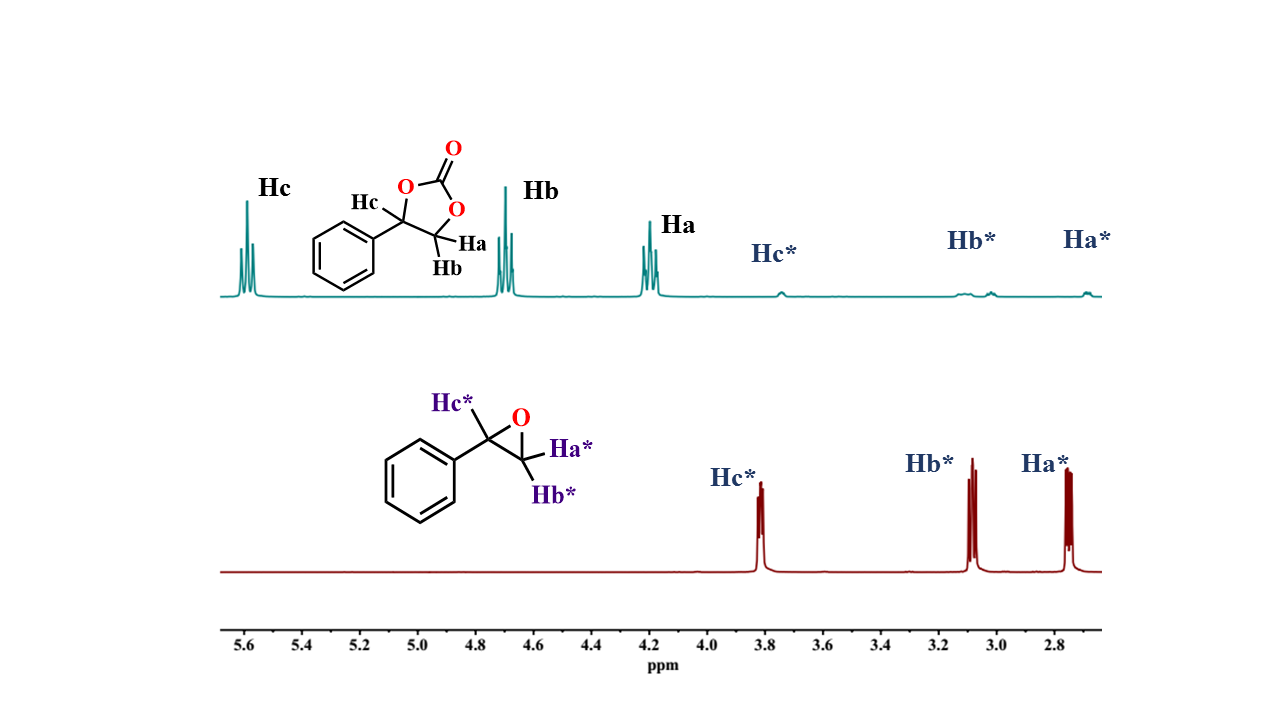
**

**Figure S13.** ^1^H NMR (CDCl_3_, 400 MHz) spectra for the cycloaddition reaction of styrene oxide with CO_2_ catalyzed by **HMP-TAPA** (Table 2, entry no. 9).

**
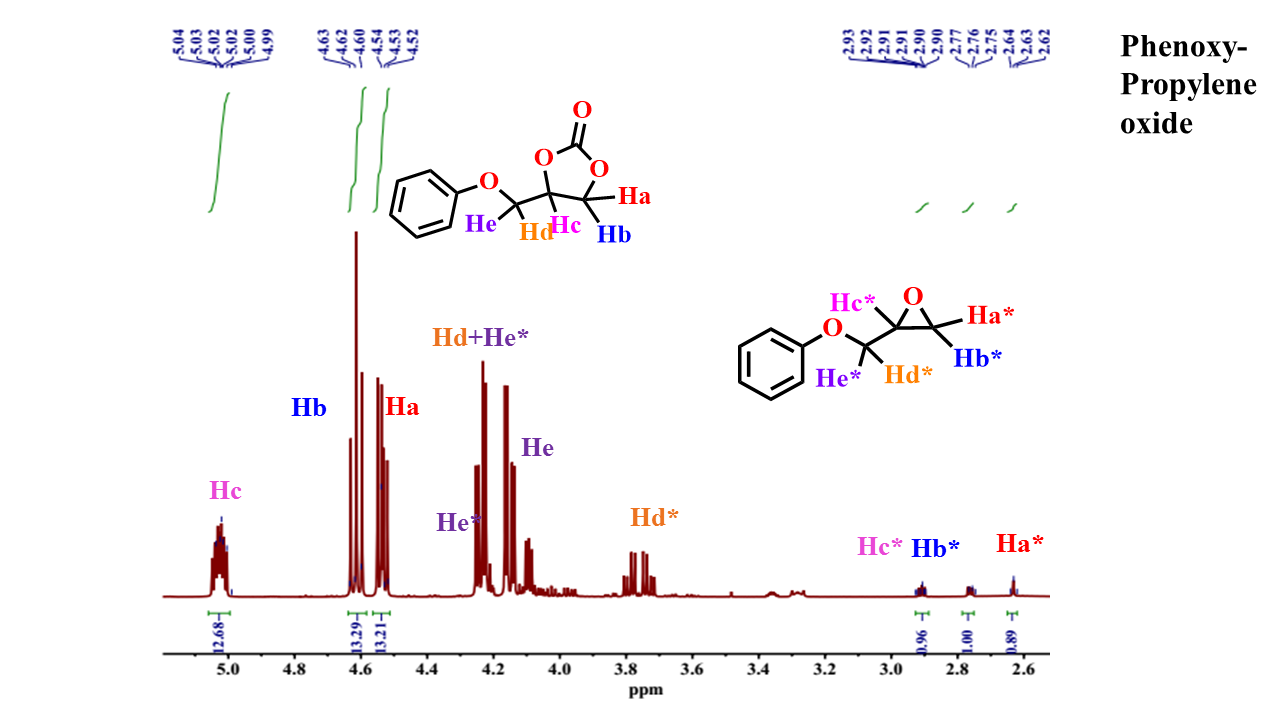
**

**Figure S14.** ^1^H NMR (CDCl_3_, 400 MHz) spectra for the cycloaddition reaction of phenyl glycidyl ether with CO_2_ catalyzed by **HMP-TAPA** (Table 2, entry no. 10).


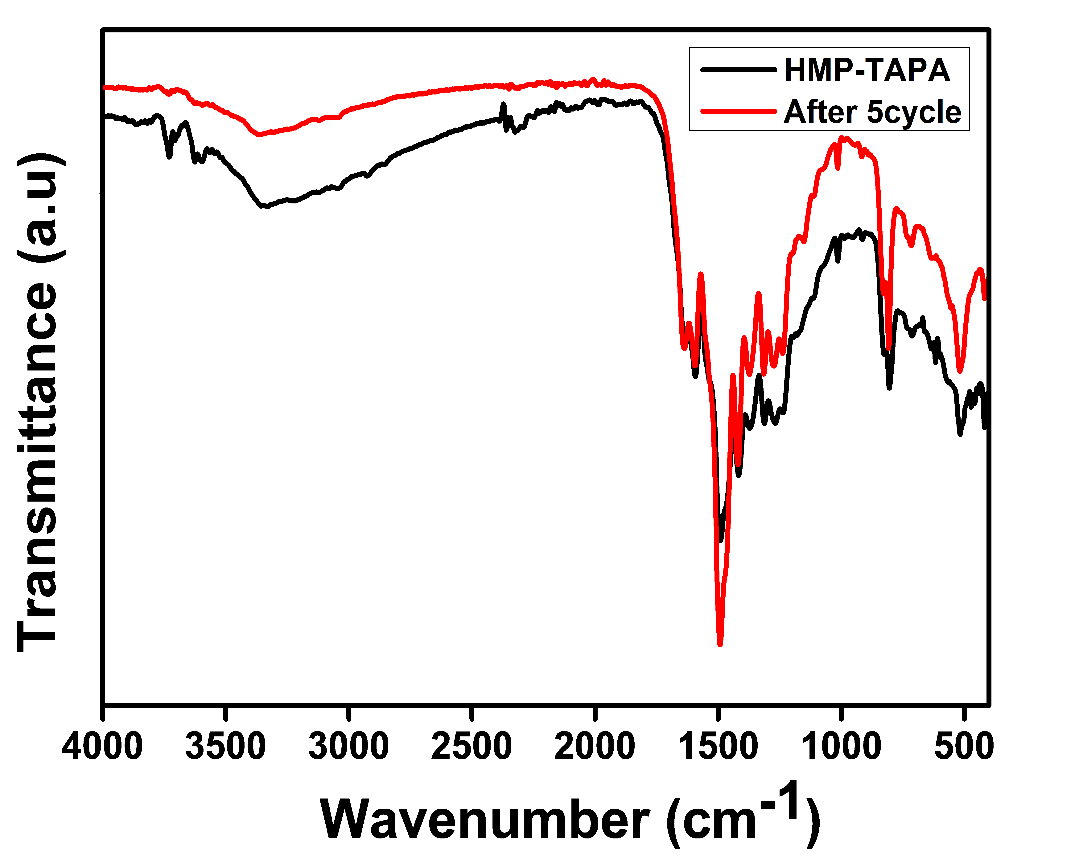


**Figure S15.** FT-IR spectra for **HMP-TAPA** and the recovered sample after five catalytic cycles.

**Table S1**. Comparison of the reaction conditions used for cycloaddition of CO_2_ by various triazine based framework taking epichlorohydrin as model substrate.

| **Sr. No.** | **Catalyst/Triazine Frameworks** | **Catalyst Loading [mg]** | **Pressure [MPa]** | **Time**  **[h]** | **Temp.**  **[^o^ C]** | **Con.**  **(%)** | **Ref.** |
| --- | --- | --- | --- | --- | --- | --- | --- |
| 1. | CTF-1 | 55 | 0.69 | 4 | 130 | 77 | [**^1^**](#_ENREF_1) |
| 2. | CTF-P-HAS | 55.5 | 0.69 | 4 | 130 | 95 | [**^1^**](#_ENREF_1) |
| 3. | CTF-0 | 55.5 | 0.69 | 4 | 130 | 93 | [**^2^**](#_ENREF_2) |
| 4. | 2,5-DCP-CTF | 55.5 | 0.69 | 4 | 130 | 95 | [**^3^**](#_ENREF_3) |
| 5. | CTF-CSU-19 | 10 | 0.1 | 48 | 25 | 96 | [**^4^**](#_ENREF_4) |
| 6. | cCTF-500 | 37 | 1.0 | 12 | 90 | 95 | [**^5^**](#_ENREF_5) |
| 7. | CCTFs-350 | 15 | 0.1 | 24 | 120 | 95 | [**^6^**](#_ENREF_6) |
| 8. | Co-PCCTFs | 15 | 0.1 | 24 | 120 | 94 | [**^7^**](#_ENREF_7) |
| 9. | NHC-CTFs | 23 | 0.5 | 6 | 100 | 97 | [**^8^**](#_ENREF_8) |
| 10. | CYA-ANIS+ TBAI | 100 | 0.1 | 12 | 105 | 80 | [**^9^**](#_ENREF_9) |
| 11. | HMP-TAPA | 10 | 0.6 | 6 | 80 | >99 | **Present Work** |

**References:**

1. J. Roeser, K. Kailasam and A. Thomas, *ChemSusChem*, 2012, **5**, 1793-1799.

2. P. Katekomol, J. Roeser, M. Bojdys, J. Weber and A. Thomas, *Chem. Mater.*, 2013, **25**, 1542-1548.

3. Y.-M. Li, L. Yang, L. Sun, L. Ma, W.-Q. Deng and Z. Li,  *J. Mater. Chem. A*, 2019, **7**, 26071-26076.

4. W. Yu, S. Gu, Y. Fu, S. Xiong, C. Pan, Y. Liu and G. Yu, *J. Catal.*, 2018, **362**, 1-9.

5. O. Buyukcakir, S. H. Je, S. N. Talapaneni, D. Kim and A. Coskun, *ACS Appl. Mater. Interfaces*, 2017, **9**, 7209-7216.

6. T.-T. Liu, R. Xu, J.-D. Yi, J. Liang, X.-S. Wang, P.-C. Shi, Y.-B. Huang and R. Cao, *ChemCatChem*, 2018, **10**, 2036-2040.

7. Q.-J. Wu, M.-J. Mao, J.-X. Chen, Y.-B. Huang and R. Cao, *Catal. Sci. Technol.*, 2020, **10**, 8026-8033.

8. C. Yue, W. Wang and F. Li, *ChemSusChem*, 2020, **13**, 5996-6004.

9. T. Biswas, A. Halder, K. S. Paliwal, A. Mitra, G. Tudu, R. Banerjee and V. Mahalingam, *Chem. Asian J.*, 2020, **15**, 1683-1687.
